# Over‐shedding of donor‐derived cell‐free DNA at immune‐related regions into plasma of lung transplant recipient

**DOI:** 10.1002/ctm2.622

**Published:** 2022-01-12

**Authors:** Jiaqi Luo, Liping Liu, Lingxi Chen, Xin Xu, Yanfei Wang, Bing Wei, Chunrong Ju, Xuedong Wang, Liyan Huang, Wenchuang Zeng, Xinyao Miao, Ling Sang, Danxia Huang, Guangze Pan, Guilin Peng, Zhuxing Chen, Zicheng Zhao, Chao Yang, Weixue Cui, Wenxi Jiang, Jinjin Xu, Shuai Cheng Li, Jianxing He

**Affiliations:** ^1^ National Clinical Research Center for Respiratory Disease The First Affiliated Hospital of Guangzhou Medical University Guangzhou China; ^2^ Department of The Translational Medicine Laboratory The First Affiliated Hospital of Guangzhou Medical University Guangzhou China; ^3^ City University of Hong Kong Shenzhen Research Institute Shenzhen China; ^4^ Department of Thoracic Surgery The First Affiliated Hospital of Guangzhou Medical University Guangzhou China; ^5^ Department of Respiratory and Critical Care Medicine Department of lung transplantation The First Affiliated Hospital of Guangzhou Medical University Guangzhou China; ^6^ Department of Critical Care Medicine The First Affiliated Hospital of Guangzhou Medical University Guangzhou China; ^7^ Department of Cardiac Surgery The First Affiliated Hospital of Guangzhou Medical University Guangzhou China; ^8^ BGI Genomics BGI‐Shenzhen Shenzhen China

Dear editor,

The discovery of the positive correlation between the fraction of donor‐derived cell‐free DNA (cfDNA) in the recipient's plasma (herein denoted as donor DNA fraction) and the risk of organ transplant rejection has empowered the development of non‐invasive methods for the prediction and prevention of organ transplant failure.^1^, ^2^ However, as previous studies mainly focused on a global estimation of donor DNA fraction and usually have been conducted months post‐transplant, some important questions remained unanswered. For example, whether the released donor DNA is an even distribution of the graft genome; if otherwise there exists certain levels of over‐representation, what biological insights are underlined; and how early a signal indicative of poor prognosis and potential needs for clinical interventions may occur.

To address the above questions, we examined in depth the cfDNA of 15 plasma samples (denoted as Dx samples) from three lung transplant recipients at multiple time points (Day 1/4/7/10/13) during the first 2 weeks post‐transplant, plus their genomic DNA obtained pre‐transplant (D0 samples), using deep (≈50X) whole genome sequencing (Figure 1A). We estimated the global donor DNA fraction for each transplant recipient at each time point based on genome‐wide SNP genotyping^1^, ^2^ (Supporting Information Methods; Figure 1B). Consistent with previous findings, donor DNA fraction peaked immediately after transplantation (day 1) and fell quickly (by day 4). Interestingly, after the sharp decrease at day 4, one of the recipient, patient 3, showed an acute relapse during days 10–13, while patients 1 and 2 showed flattening/slow‐decreasing trends. This early dynamics was in line with patient outcome (Table 1): patient 3 was detected positive of anti‐HLA‐II antibodies at 26 days post‐transplant and developed pleural effusion, a sign of acute lung injury at 5 weeks post‐transplant; while patients 1 and 2 showed no signs of allograft dysfunction 18 months post‐transplant.

**FIGURE 1:**
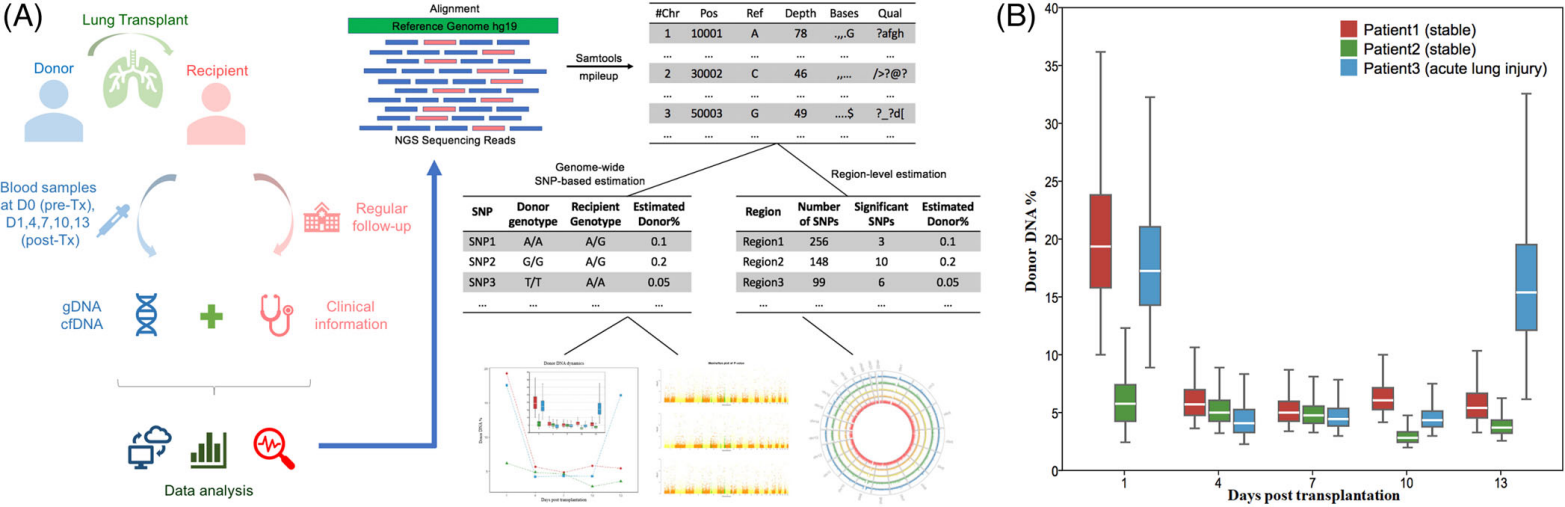
(A) Study design and data analysis flowchart. Blood samples were collected at day 0 (pre‐transplant), and days 1,4,7,10,13 (post‐transplant) from recipients of lung transplantation (LTx). Genomic DNA (gDNA) and cell‐free DNA (cfDNA) were extracted from the blood samples and subjected to high coverage whole‐genome sequencing. Sequencing data were analysed together with follow‐up clinical information. A global donor DNA fraction is estimated for each patient at each time point. Over‐representation of graft DNA was examined at SNP‐level and region‐level. See Supporting Information Methods for a full description of data processing and analysis. (B) Dynamics of global donor DNA fraction during the first 2 weeks after LTx. Global donor DNA fraction of the three patients at days 1, 4, 7, 10, 13 (post‐transplant) were predicted using the genome‐wide SNP‐based method. Detailed values of global donor DNA fraction for all samples can be found in Appendix S1

**TABLE 1:** 

Patient no.	Sample no.	Time point post transplant surgery (day)	Gender	Age	Anti‐rejection scheme	White bood cell count (10E9/L)	Lymphocyte count (10E9/L)	Lymphocyte percentage	Follow‐up
Patient 1	Patient 1_D‐1	‐1	Male	59	‐	13	2	15.2	anti‐HLA tests all negative; no apparent signs of allograft dysfunction 18 months post‐transplant
	Patient 1_D1	1			Sou‐Medrol/KF506/MMF/Basiliximab	16.6	0.6	3.4	
	Patient 1_D4	4			Sou‐Medrol/KF506/Basiliximab	13.1	0.3	2.4	
	Patient 1_D7	7			Sou‐Medrol/KF506	9.32	0.2	1.9	
	Patient 1_D10	10			Sou‐Medrol/KF506	12.1	0.5	4	
	Patient 1_D13	13			KF506/MPIV	11.3	0.6	5.6	
Patient 2	Patient 2_D‐1	‐1	Female	45	‐	3.9	0.7	18.8	anti‐HLA tests all negative; no apparent signs of allograft dysfunction 18 months post‐transplant
	Patient 2_D1	1			Sou‐Medrol/KF506/MMF	8.4	0.3	3.7	
	Patient 2_D4	4			Sou‐Medrol/KF506/MMF	8.2	0.5	6.3	
	Patient 2_D7	7			Sou‐Medrol/KF506/MMF	10.01	0.1	0.8	
	Patient 2_D10	10			Sou‐Medrol/KF506/MMF	6.29	0	0.6	
	Patient 2_D13	13			Sou‐Medrol/KF506/MMF	6	0.2	3.5	
Patient 3	Patient 3_D‐1	‐1	Male	60	‐	5.6	1.6	28.5	anti‐HLA‐II positive at 26‐days post‐transplant; pleural effusion 5‐weeks post‐transplant; multiple admission within the first year post‐transplant
	Patient 3_D1	1			Sou‐Medrol/KF506/MMF	14.3	0.6	4.2	
	Patient 3_D4	4			Sou‐Medrol/MMF	7.5	0.6	8.7	
	Patient 3_D7	7			Sou‐Medrol/MMF	7.8	0.6	7.9	
	Patient 3_D10	10			KF506/MMF	10.6	0.8	8	
	Patient 3_D13	13			KF506/MMF	12.48	0.6	5.2	

Next, we asked if there were any regions of donor‐derived cfDNA over‐represented in the recipients’ plasma. We first examined at a region‐level by splitting each chromosome into 500 kb‐windows and used a maximum‐likelihood‐based method^3^ (Supporting Information Methods) to estimate a donor DNA fraction for each window (Figures S1–S3; Additional Table S2). *p*‐Value for each window was calculated assuming a normal distribution. We found 0.2% (13/5435) of the windows significantly (FDR ≤ .1) over‐represented in all three patients, mainly distributed at 1p36, 1q21, 9p12‐13, 20q11 and 21q11 (Appendix S2). We noticed some overlap of these regions with previously reported regions of structural complexity.^4^ However, the over‐representation was not likely to be caused by copy number variations (duplications) in the Dx samples because the levels of duplication in D0 samples were higher at these significant regions compared to Dx samples, and the absence of significant regions where higher levels of duplication occurred in Dx versus D0 samples (Figure S4).

We then examined over‐representation at individual SNP‐levels. We deduced the donor fraction β_i_ for each selected SNP_i_ and calculated *p*‐value for each β_i_ (Supporting Information Methods; Additional Table S3). Significantly over‐represented SNP was determined as being called in all three patients with FDR ≤ .1. Figure 2A shows log *p*‐value (adjusted) versus chromosomal position of genome‐wide SNPs for the three recipients. We identified three significant regions, namely chr6:30782303‐31426881, chr7:75883092‐77138957 and chr8:7237702‐7978545, which were consistently enriched with over‐represented SNPs in all three recipients. Significant regions were defined as having ≥ 5 significant SNPs, each being less than 500 kb apart from its closest significant SNPs (Figure 2A,B). Interestingly, chr6:30782303‐31426881 overlapped with the human leukocyte antigen (HLA) region, which includes a series of immune‐related genes such as HLA‐A/B/C, MICA, DDR1; chr8:7237702‐7978545overlapped with the family of β defensin genes, for example, DEFB103/104/105/106.

**FIGURE 2:**
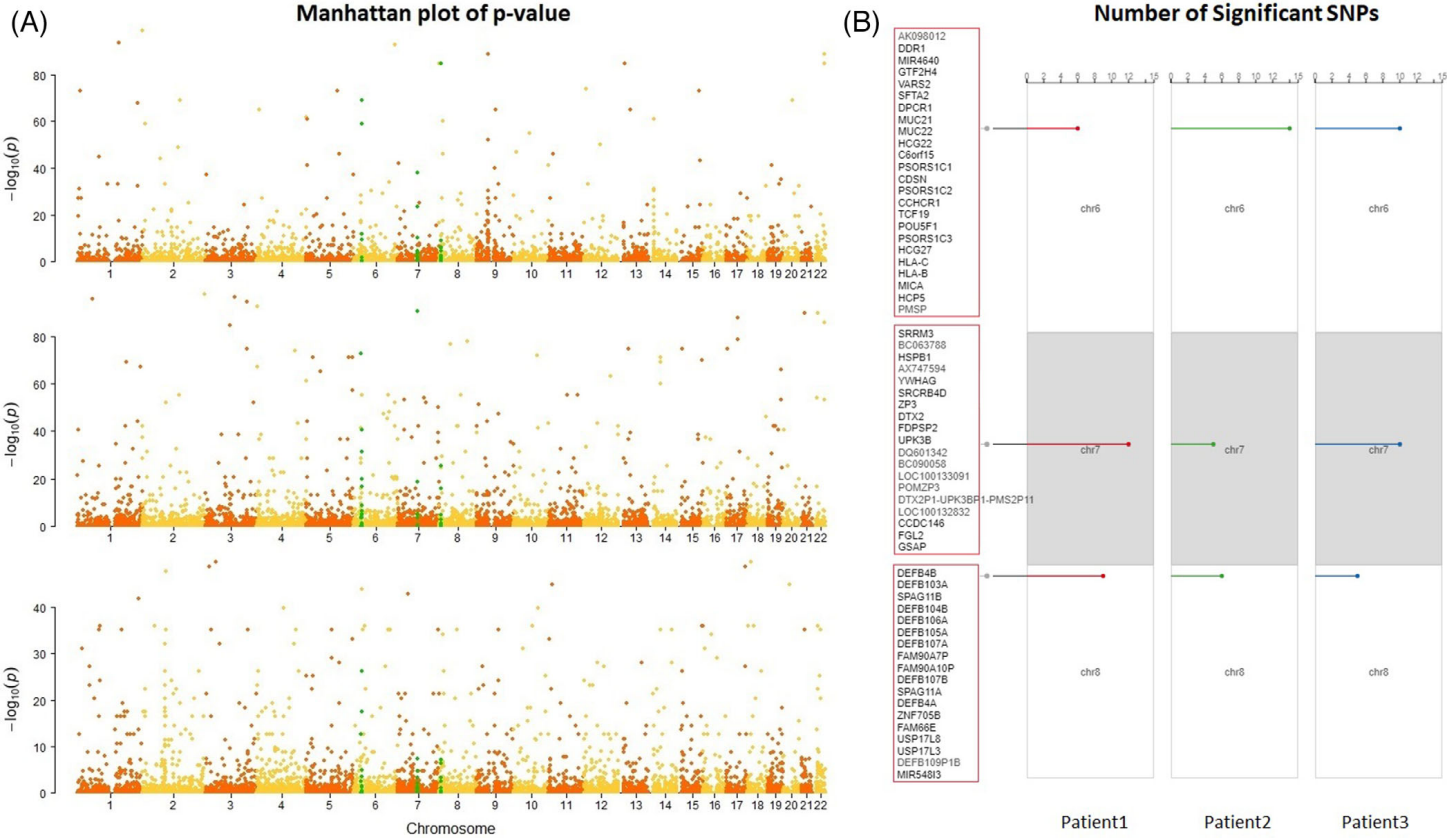
(A) Manhattan plot of median (out of five time points for each patient) log adjusted *p*‐values versus chromosomal positions of all SNPs for each of the three patients. The regions consistently enriched with significantly over‐represented SNPs in all three patients are highlighted in green colour; the 22 chromosomes are coloured yellow/orange in turn. (B) The three regions enriched with significantly over‐represented SNPs: chr6:30782303‐31426881, chr7:75883092‐77138957 and chr8:7237702‐7978545. All genes within the region and the number of significant SNP counts for each recipient are shown. The positions where the bands stand at each chromosome are proportional to the positions of the enriched regions at the chromosome. The plotting is powered by Bio‐oviz focal cluster plot: https://bio.oviz.org/demo‐project/analyses/focal‐cluster‐r

We then retrieved all genes within the enriched regions and performed enrichment analysis^5^ to identify significant KEGG pathways and biological processes (*p* ≤ .05; Figure 3A). The results revealed strong associations with graft–host immune responses and antimicrobial activities.

**FIGURE 3:**
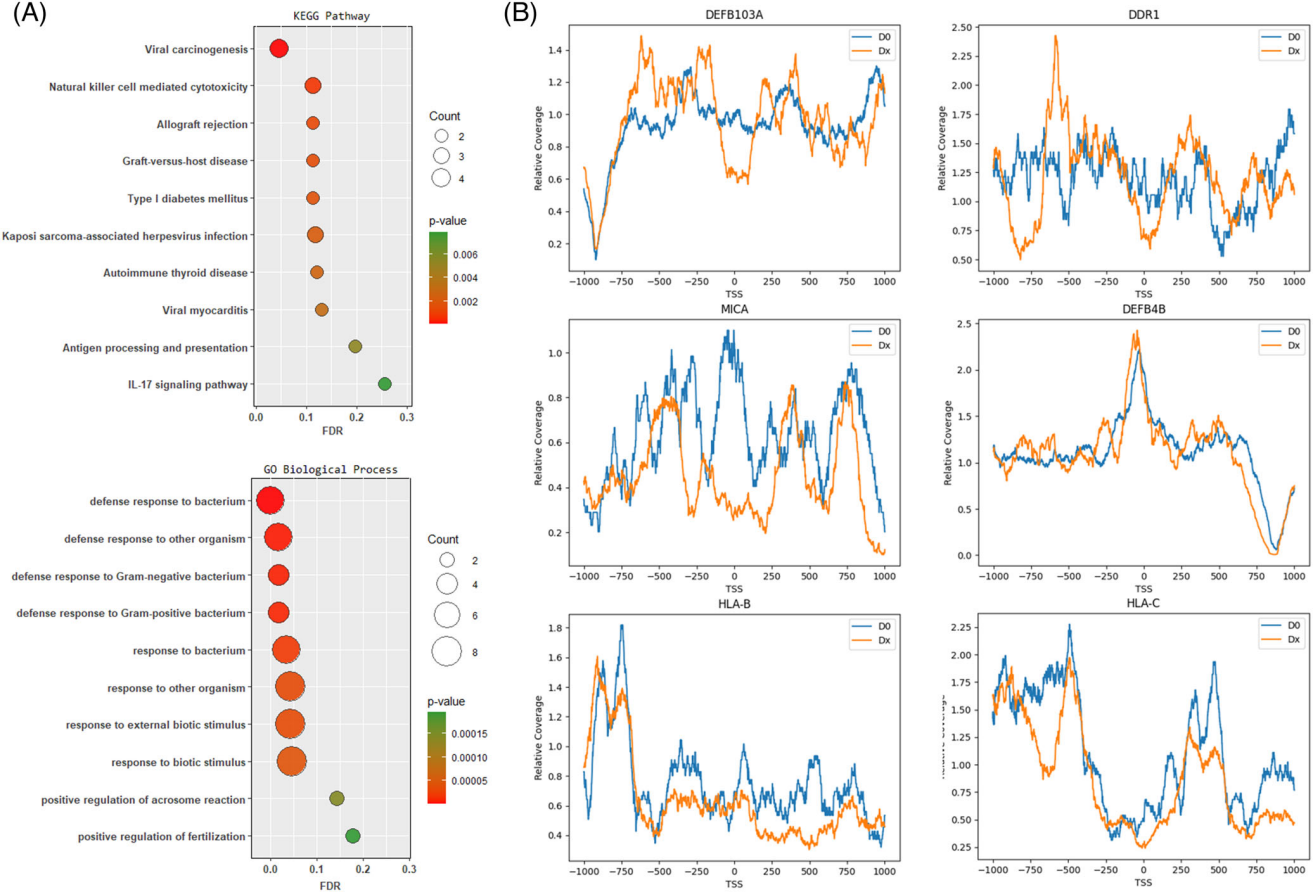
(A) Top KEGG pathways and GO biological processes enriched (*p* ≤ 0.05) by genes within the three significant regions. Colours and sizes of the bubbles correspond to *p*‐value and the number of query genes mapped to the pathway or process. (B) Nucleosome footprints of selected genes within the over‐represented immune‐related regions. Blue curves showed relative coverage of the D0 (gDNA) sample averaged over the three patients; orange curves showed the relative coverage as an average of 15 Dx (cfDNA) samples. Note that the plots are drawn from relatively small number of samples; the curves are noisy

To further investigate the causes of the “over‐shedding”, we computed nucleosome footprints^6^ of genes within the significant regions pre‐ and post‐transplant. Characteristics of open chromatin were found in some of the genes post‐transplant, suggesting active transcriptions (Figure 3B). Among these likely active genes, some are known to be expressed in lung epithelial during inflammation and transplantation, for example, DEFB103, DDR1 and MICA^7^, ^8^, ^9^; some known to be expressed in both the host and the graft, for example, HLA‐B/C. Interestingly, DEFB103 and DDR1 which are expected to be expressed only in the graft showed different nucleosome footprints pre‐ and post‐transplant, while genes known to expressin both, for example, HLA‐B/C, preserved the shape of the footprints.

Actively transcribed regions are considered more likely to have open chromatin structures, which in turn leads to more nuclease‐mediated degradation.^6^ It is believed that the major source of plasma cfDNA is dying cells resulted from apoptosis/necrosis. A great lymphocyte turnover post‐transplant can be inferred from the white blood cell count (Additional Table S1), indicating the host lymphocytes as the major source of host cfDNA. Relatively high gene expression in these cells can result in the seemingly “over‐shedding” phenomenon—it is in fact the “over‐degradation” of host cfDNA at these regions, rather than the “over‐shedding” of graft cfDNA. However, for genes more actively or only expressed in the graft, there should be extra sources of cfDNA release, for example, active secretion via extracellular vesicles,^10^ because otherwise they would rather appear “under‐shedding”.

There are several limitations. The small cohort size has affected the confidence of the results and the interpretations. Although our results confirmed the previously published prognostic value of cfDNA monitoring, a larger cohort with comprehensive clinical data will be necessary to establish the role of early donor DNA dynamics on post‐transplant manipulations. The clinical significances of the identified regions need further experimental validations, and it is still unclear what extra benefits could be drawn from testing these regions. Nevertheless, this is the first report of an uneven distribution of donor‐derived cfDNA and the over‐represented immune‐related regions, which is expected to provide insights into our understanding of cfDNA in organ transplantation.

## CONFLICT OF INTEREST

The authors declare that they have no conflict of interest.

## CONSENT FOR PUBLICATION

Informed written consent for publication was obtained from all human participants.

## Supporting information


**Supplementary Figures 1–3**. Circos plots of estimated regional donor DNA fraction on 500kb windows for each recipient at each time point. Time points are arranged from inside to outside ‐ the innermost circle is day 1, and the outermost circle is day 13. The heights of the bars are proportional to the values of the estimated donor DNA fraction. Blank regions indicate lack of informative SNPs at the corresponding window. The circos plot is powered by Bio‐oviz circos plot tool: https://bio.oviz.org/demo‐project/analyses/Circos.
**Supplementary Figure 4**. Mean depth of SNPs at each 500kb window of chromosome 1 in Dx and D0 samples. Significantly over‐represented windows are highlighted in red boxes. Previously reported regions of structural complexity (main text Ref 4) are highlighted in yellow boxes.
**Additional Table 1**. Patient demographics and clinical assay data.
**Additional Table 2**. Regional donor DNA fraction for each patient at each time point with p‐value for each region.
**Additional Table 3**. Deducted donor fraction value for genome‐wide SNPs for each patient at each time point and median p‐value for each SNP.
**Appendix 1**. Predicted global donor DNA fraction for each patient at each time point.
**Appendix 2**. 500kb‐window regions with significant graft DNA over‐representation
