# Supplementary material for: Over‐shedding of donor‐derived cell‐free DNA at immune‐related regions into plasma of lung transplant recipient
Source: Clin Transl Med. 2022 Jan 12;12(1):e622. doi: 10.1002/ctm2.622 (PMC8754174; doi:10.1002/ctm2.622)
Supplement: Supplementary file 1 — Supplementary Figures 1–3. Circos plots of estimated regional donor DNA fraction on 500kb windows for each recipient at each time point. Time points are arranged from inside to outside ‐ the innermost circle is day 1, and the outermost circle is day 13. The heights of the bars are proportional to the values of the estimated donor DNA fraction. Blank regions indicate lack of informative SNPs at the corresponding window. The circos plot is powered by Bio‐oviz circos plot tool: https://bio.oviz.org/demo‐project/analyses/Circos. Supplementary Figure 4. Mean depth of SNPs at each 500kb window of chromosome 1 in Dx and D0 samples. Significantly over‐represented windows are highlighted in red boxes. Previously reported regions of structural complexity (main text Ref 4) are highlighted in yellow boxes. Additional Table 1. Patient demographics and clinical assay data. Additional Table 2. Regional donor DNA fraction for each patient at each time point with p‐value for each region. Additional Table 3. Deducted donor fraction value for genome‐wide SNPs for each patient at each time point and median p‐value for each SNP. Appendix 1. Predicted global donor DNA fraction for each patient at each time point. Appendix 2. 500kb‐window regions with significant graft DNA over‐representation [file CTM2-12-e622-s001.docx]

**Supplementary Methods**

**Patients and Sample collection**

Three patients who have received lung transplantation (LTx) at The First Affiliated Hospital of Guangzhou Medical University were included in this study. Whole blood samples were drawn from each LTx recipient at Day 0 (denoted as D0 sample; pre-transplant), and Day 1, 4, 7, 10, 13 (denoted as Dx samples; post-transplant). Hence for each of the three patient we have a D0 sample for recipient genotyping and five Dx samples containing donor-derived cfDNA (Figure 1A).

**DNA extraction, library preparation and whole-genome sequencing**

Genomic DNA (gDNA) was extracted from D0 samples and cfDNA was extracted from Dx samples. In brief, purified gDNA from D0 samples was extracted using QIAamp DNA Blood Mini Kit (Qiagen, Germen); cfDNA from Dx samples was extracted using the QIAamp Circulating Nucleic Acid Kit (Qiagen, Germen), according to the manufacturer’s instructions, separately.

Library preparation was performed with MGIEasy Universal DNA Library Prep Set and Customer self-developed kit (MGI, Shenzhen, China). For gDNA, we fragmented it to ~250bp insert size length. We performed a non-tempplated dAMP onto the 3’ end of the fragments, which is known as dA-tailing. As for cfDNA, which is only ~170bp, we performed end repair and dA-tailing processes. Then we performed adapter ligation and Pre-PCR, to obtain high-quality libraries before sequencing. DNA quantification was performed with Qubit 3.0 (Thermo Fisher Scientific, USA). DNB preparation kits and Sequencing kits (MGI, Shenzhen, China) were used to pumped DNBs (DNA nanoballs) and load the DNBs onto the Patterned Array chip. Pair-end, 150bp sequencing was performed using MGISEQ-2000 with an expected amount of data >150Gb per sample.

**Data pre-processing**

Raw sequencing reads were subjected to quality check and filtering using Fastp [1]. Read mapping to human genome hg19 was performed using BWA MEM with the command “bwa mem -t 8 -M” [2]. Duplicates were removed and local realignment was performed using the GATK bundle [3]. Genotypes (pileup reads) of the D0 and Dx samples on all positions were determined using the mpileup function of Samtools [4]. SNPs were called if the read genotype(s) differ between D0 and Dx samples (Figure 1A).

**Genome-wide SNP-based global donor DNA fraction estimation**

Genome-wide SNPs (excluding sex chromosomes) that have a strictly homozygous genotype in the D0 (gDNA) sample and a different genotype in the Dx (cfDNA) samples were first selected. To reduce the effects of rare genetic variants on reproducibility, variants with a gnomAD_ALL population frequency lower than 0.3 were filtered [5]. For each of the selected SNPs, the minor allele frequency (MAF) was calculated [6, 7]. We consider the global donor DNA fraction as an average of all observed values of donor fraction (herein denoted as β) deducted from each selected SNP_i_. We assume that each β_i_ follows normal distribution. If the donor genotype on a given SNP_i_ is also homozygous (i.e., wildtype/wildtype in recipient and mutant/mutant in donor or the other way around), β_i_ is equal to MAF; if the donor genotype is heterozygous, β_i_ will equal two times MAF (i.e., half of the donor fraction equals MAF). Therefore, the list of MAFs is a mixture of two normal distributions, one centred at 0.5β and the other centred at β. We then calculate the average of the list (denoted as x), we have 0.5β < x < β, which transforms into

$$0.5x < 0.5\beta,$$

$$\beta< 2x.$$

Using the above constraints, we first filter out outliers beyond the range of (0.5x, 2x). Next, to separate values from the two different distributions, we sorted the list, and found the best separating value point that minimizes the total intra-group variance, which can be described as the following objective function.

$$\sum_{i=1}^{2} \sum_{x\in S_{i}} \left\| x - \mu_{i} \right\|^{2}$$

This is known as the objective function of k-means clustering when k=2. However, k-means clustering does not guarantee a global optimum (can be trapped into local optima), while a greedy algorithm can achieve global optima when k=2, with time complexity O(n). All values in the sorted list before the best separating value point are considered as samples of the 0.5β distribution, these values are then multiplied by 2. The mean of the resulted new list of values is the predicted β, i.e., the predicted global donor DNA fraction.

**Region-level donor DNA fraction estimation and over-representation analysis**

The above genome-wide estimation method is considered not suitable for calculating donor DNA fraction in a small region (e.g. 500kb), because there could be too few SNPs that satisfy the ‘strictly homozygous genotype in D0 sample and a different genotype in the Dx samples’ condition within a given small region. Hence for region-level estimation, we utilized a method that does not require D0 to be homozygous, which allow more SNP information to be considered. This is a simplified variant of a previously published maximum likelihood-based method [8].

Maximum-likelihood-estimation (MLE) of donor DNA fraction for a small region. Assuming (under ideal conditions) each SNP has only three possible values of wildtype frequency: {0, 0.5, 1} on both the recipient and the donor. For each SNP (denoted as SNP_i_), the number of reads supporting the wildtype genotype k_i_ and the number of reads supporting the mutant genotype q_i_ are known. If given β the donor DNA fraction, then the wildtype frequency w_i_ of SNP_i_ in the cfDNA sample have 3^2^ = 9 possible values: {0, 0.5β, β, 0.5(1-β), 0.5, 0.5(1+β), 1-β, 1-0.5β, 1}. The probability p_i_ of observing k_i_ and q_i_ given wildtype frequency w_i_ for SNP_i_ can be calculated as followed.

$$p_{i}{=C}_{\left( k_{i}+q_{i} \right)}^{k_{i}}w_{i}^{k_{i}}\left( 1-w_{i} \right)^{qi} (1)$$

Then for all the SNPs, the total probability p is the products of each p_i_.

$$p=\prod_{i=1}^{n} p_{i}= \prod_{i=1}^{n} C_{\left( k_{i}+q_{i} \right)}^{k_{i}}w_{i}^{k_{i}}\left( 1-w_{i} \right)^{qi} (2)$$

$$log p=\sum_{i=1}^{n} {\log p}_{i}=\sum_{i=1}^{n} k_{i}logw_{i}+q_{i}log{(1-w}_{i}) + const(3)$$

$$\underset{0<\beta<1}{\mathrm{argmax}} \{\underset{w}{\mathrm{argmax}} \log p\} (4)$$

For a given β on a given SNP_i_, we can find out the w_i_ that gives the biggest p_i_. Repeat it to all SNPs, we can calculate the maximum log *p* for the given β. Different choices of β values yield different maximum log *p* (i.e., log-likelihood). By iterating through evenly spaced numbers within a specified interval of possible β values, the method can find out the closest approximation of the best β resulting in a maximum log-likelihood.

Unlike the global method which uses only SNPs with homozygous genotypes in D0, the regional method starts with all SNPs. To reduce the effects of rare genetic variants on reproducibility while keeping as much as SNPs as possible for the calculations, variants with a gnomAD_ALL population frequency lower than 0.01 were filtered. We split the 22 autosomes into 500kb windows and estimated the donor DNA fraction for each region at each time point. Last window of each chromosome is less than 500kb; windows with no informative SNPs (i.e., differed D0 and Dx genotypes) were removed. For each transplant recipient, a circos plot of estimated regional donor DNA fraction was drawn for all five time points (Supplementary Figures 1-3). Assuming normal distribution of the regional donor DNA fraction values, we calculated p-values for all windows at each time point and corrected the median p-values for multiple tests using Benjamini-Hochberg adjustment. Over-represented regions are defined as having an FDR ≤ 0.1 for each transplant recipient.

**SNP-level over-representation analysis**

From above genome-wide method we estimated donor fraction β_i_ for each selected SNP_i_. We then calculated a p-value for each β_i_ assuming normal distribution and corrected the median p-values for multiple tests using Benjamini-Hochberg adjustment (Additional Table 3). We defined significantly over-represented SNP as having an FDR ≤ 0.1 for each transplant recipient. A Manhattan plot of median log adjusted p-value of all selected SNPs over the genome (excluding sex chromosomes) was drawn for each recipient using the qqman R package [9] (Figure 2A). We further defined regions with enrichment of significantly over-represented SNPs as regions satisfying: (1) contain ≥ 5 significant SNPs, and (2) each significant SNP is less than 0.5M apart from its nearest significant SNPs. The identified enriched regions that overlapped in all recipients are highlighted in green colour on the Manhattan plots.

**Functional enrichment analysis**

Genes within the identified enriched regions that overlapped in all recipients were retrieved using the UCSC table browser [10] (Appendix 3). Enrichment of KEGG pathways and GO terms of biological process were identified using the WebGestalt [11] web tool. Hits with a p-value ≤ 0.05 were plotted in Figure 3A.

**Nucleosome foot-printing**

Nucleosome foot-printing of genes of interest were performed as previously described [12, 13]. A nucleosome footprint for a gene is the relative coverage (position depth divided by average depth) at $\pm$ 1kb of the transcription start site (TSS). Here we used the mean of the three D0 samples and mean of the total 15 Dx samples as input for plotting, to visualize changes of nucleosome occupancy pre- and post-transplant.

**References**

1. Chen S, Zhou Y, Chen Y, Gu J (2018) fastp: an ultra-fast all-in-one FASTQ preprocessor. Bioinformatics 34:i884–i890. https://doi.org/10.1093/bioinformatics/bty560

2. Li H, Durbin R (2009) Fast and accurate short read alignment with Burrows-Wheeler transform. Bioinformatics 25:1754–1760. https://doi.org/10.1093/bioinformatics/btp324

3. McKenna A, Hanna M, Banks E, et al (2010) The Genome Analysis Toolkit: a MapReduce framework for analyzing next-generation DNA sequencing data. Genome Res 20:1297–303. https://doi.org/10.1101/gr.107524.110

4. Li H, Handsaker B, Wysoker A, et al (2009) The Sequence Alignment/Map format and SAMtools. Bioinformatics 25:2078–2079. https://doi.org/10.1093/bioinformatics/btp352

5. Karczewski KJ, Francioli LC, Tiao G, et al (2020) The mutational constraint spectrum quantified from variation in 141,456 humans. Nature 581:434–443. https://doi.org/10.1038/s41586-020-2308-7

6. De Vlaminck I, Valantine HA, Snyder TM, et al (2014) Circulating cell-free DNA enables noninvasive diagnosis of heart transplant rejection. Sci Transl Med 6:. https://doi.org/10.1126/scitranslmed.3007803

7. De Vlaminck I, Martin L, Kertesz M, et al (2015) Noninvasive monitoring of infection and rejection after lung transplantation. Proc Natl Acad Sci U S A 112:13336–13341. https://doi.org/10.1073/pnas.1517494112

8. Sharon E, Shi H, Kharbanda S, et al (2017) Quantification of transplant-derived circulating cell-free DNA in absence of a donor genotype. PLoS Comput Biol 13:e1005629. https://doi.org/10.1371/journal.pcbi.1005629

9. D. Turner S (2018) qqman: an R package for visualizing GWAS results using Q-Q and manhattan plots. J Open Source Softw 3:731. https://doi.org/10.21105/joss.00731

10. Kent WJ, Sugnet CW, Furey TS, et al (2002) The human genome browser at UCSC. Genome Res 12:996–1006. https://doi.org/10.1101/gr.229102

11. Liao Y, Wang J, Jaehnig EJ, et al (2019) WebGestalt 2019: gene set analysis toolkit with revamped UIs and APIs. Nucleic Acids Res 47:W199–W205. https://doi.org/10.1093/nar/gkz401

12. Snyder MW, Kircher M, Hill AJ, et al (2016) Cell-free DNA Comprises an in Vivo Nucleosome Footprint that Informs Its Tissues-Of-Origin. Cell 164:57–68. https://doi.org/10.1016/j.cell.2015.11.050

13. Ulz P, Thallinger GG, Auer M, et al (2016) Inferring expressed genes by whole-genome sequencing of plasma DNA. Nat Genet 48:1273–1278. https://doi.org/10.1038/ng.3648

**Supplementary Figures**

[Supplementary_Figures.zip](https://drive.google.com/uc?export=download&id=19mf_T-LWVqi7vBosEnDRFamJE0_WJmZ2) (click to download or use web browser to open https://drive.google.com/uc?export=download&id=19mf_T-LWVqi7vBosEnDRFamJE0_WJmZ2)

**Supplementary Figures 1-3.** Circos plots of estimated regional donor DNA fraction on 500kb windows for each recipient at each time point. Time points are arranged from inside to outside - the innermost circle is day 1, and the outermost circle is day 13. The heights of the bars are proportional to the values of the estimated donor DNA fraction. Blank regions indicate lack of informative SNPs at the corresponding window. The circos plot is powered by Bio-oviz circos plot tool: <https://bio.oviz.org/demo-project/analyses/Circos>.

**Supplementary Figure 4**. Mean depth of SNPs at each 500kb window of chromosome 1 in Dx and D0 samples. Significantly over-represented windows are highlighted in red boxes. Previously reported regions of structural complexity (main text Ref 4) are highlighted in yellow boxes.

**Additional Tables**

[Additional_Tables_1-3.zip](https://drive.google.com/uc?export=download&id=1QVUhucbJapbhZ12PMJ9qrfyCWH97tEM-) (click to download or use web browser to open https://drive.google.com/uc?export=download&id=1QVUhucbJapbhZ12PMJ9qrfyCWH97tEM-)

**Additional Table 1.** Patient demographics and clinical assay data.

**Additional Table 2**. Regional donor DNA fraction for each patient at each time point with p-value for each region.

**Additional Table 3**. Deducted donor fraction value for genome-wide SNPs for each patient at each time point and median p-value for each SNP.

**Appendices**

**Appendix 1**. Predicted global donor DNA fraction for each patient at each time point.

| Sample | Global Donor DNA % |
| --- | --- |
| Patient1_D1 | 19.61% |
| Patient1_D4 | 5.88% |
| Patient1_D7 | 5.06% |
| Patient1_D10 | 6.07% |
| Patient1_D13 | 5.67% |
| Patient2_D1 | 6.34% |
| Patient2_D4 | 5.10% |
| Patient2_D7 | 4.76% |
| Patient2_D10 | 2.85% |
| Patient2_D13 | 3.66% |
| Patient3_D1 | 17.73% |
| Patient3_D4 | 4.35% |
| Patient3_D7 | 4.50% |
| Patient3_D10 | 4.39% |
| Patient3_D13 | 16.52% |

**Appendix 2**. 500kb-window regions with significant graft DNA over-representation

| chrom | start | end | band |
| --- | --- | --- | --- |
| chr1 | 17000001 | 17500000 | 1p36.13 |
| chr1 | 143500001 | 144000000 | 1q21.1 |
| chr1 | 144000001 | 144500000 | 1q21.1 |
| chr1 | 144500001 | 145000000 | 1q21.1 |
| chr1 | 145000001 | 145500000 | 1q21.1 |
| chr1 | 148500001 | 149000000 | 1q21.2 |
| chr1 | 149500001 | 150000000 | 1q21.2 |
| chr9 | 39500001 | 40000000 | 9p13.1 |
| chr9 | 42000001 | 42500000 | 9p12 |
| chr9 | 42500001 | 43000000 | 9p12 |
| chr9 | 43000001 | 43500000 | 9p12 |
| chr20 | 29000001 | 29500000 | 20q11.1-21 |
| chr21 | 14000001 | 14500000 | 21q11.1-2 |
